# Phase II, randomized, placebo-controlled study of dovitinib in combination with fulvestrant in postmenopausal patients with HR^+^, HER2^−^ breast cancer that had progressed during or after prior endocrine therapy

**DOI:** 10.1186/s13058-017-0807-8

**Published:** 2017-02-10

**Authors:** Antonino Musolino, Mario Campone, Patrick Neven, Neelima Denduluri, Carlos H. Barrios, Javier Cortes, Kimberly Blackwell, Hatem Soliman, Zsuzsanna Kahan, Hervé Bonnefoi, Matthew Squires, Yong Zhang, Stephanie Deudon, Michael M. Shi, Fabrice André

**Affiliations:** 1 Medical Oncology Unit, Azienda Ospedaliero-Universitaria di Parma, Via Gramsci 14, 43126 Parma, Italy; 2 Institut de Cancerologie de l’Ouest, René Gauducheau, Saint-Herblain, France; 3 Hospital Gasthuisberg, Leuven, Belgium; 4 Virginia Cancer Specialists, US Oncology Research, Arlington, VA USA; 5 Pontificia Universidade Católica do Rio Grande do Sul School of Medicine, Porto Alegre, Brazil; 6 Vall d’Hebron Institute of Oncology, Barcelona, Spain; 7 Ramon y Cajal University Hospital, Madrid, Spain; 8 Duke University Medical Center, Durham, NC USA; 9 Moffitt Cancer Center, Tampa, FL USA; 10 University of Szeged, Szeged, Hungary; 11 Institut Bergonié Comprehensive Cancer Centre, Université de Bordeaux, Bordeaux, France; 12 Novartis Pharma AG, Basel, Switzerland; 13 Novartis Pharmaceuticals Corporation, East Hanover, NJ USA; 14 Institut Gustave Roussy, Villejuif, France

**Keywords:** Dovitinib, TKI285, Fulvestrant, FGF, FGFR, Breast cancer, Endocrine resistance

## Abstract

**Background:**

Overexpression of fibroblast growth factor receptor 1 (FGFR1), found in ≤8% of hormone receptor–positive (HR^+^), human epidermal growth factor receptor 2–negative (HER2^−^) breast cancer cases, is correlated with decreased overall survival and resistance to endocrine therapy (ET). Dovitinib, a potent FGFR inhibitor, has demonstrated antitumor activity in heavily pretreated patients with *FGFR* pathway–amplified breast cancer.

**Methods:**

In this randomized, placebo-controlled phase II trial, we evaluated whether the addition of dovitinib to fulvestrant would improve outcomes in postmenopausal patients with HR^+^, HER2^−^ advanced breast cancer that had progressed during or after prior ET. Patients were stratified by *FGF* pathway amplification and presence of visceral disease, and they were randomized 1:1 to receive fulvestrant plus dovitinib or placebo. The primary endpoint was progression-free survival (PFS).

**Results:**

From 15 May 2012 to 26 November 2014, 97 patients from 36 centers were enrolled. The frequency of *FGF* pathway amplification was lower than anticipated, and the study was terminated early owing to slow accrual of patients with *FGF* pathway amplification. The median PFS (95% CI) was 5.5 (3.8–14.0) months vs 5.5 (3.5–10.7) months in the dovitinib vs placebo arms, respectively (HR, 0.68; did not meet predefined efficacy criteria). For the *FGF* pathway–amplified subgroup (*n* = 31), the median PFS (95% CI) was 10.9 (3.5–16.5) months vs 5.5 (3.5–16.4) months in the dovitinib vs placebo arms, respectively (HR, 0.64; met the predefined superiority criteria). Frequently reported adverse events in the dovitinib (diarrhea, nausea, vomiting, asthenia, and headache) and placebo (diarrhea, fatigue, nausea, and asthenia) arms were mostly low grade.

**Conclusions:**

The safety profile of dovitinib plus fulvestrant was consistent with the known safety profile of single-agent dovitinib. Dovitinib in combination with fulvestrant showed promising clinical activity in the *FGF* pathway*–*amplified subgroup. However, the data reported herein should be interpreted with caution, given that fewer PFS events occurred in the *FGF* pathway–amplified patients than was expected and that an effect of dovitinib regardless of *FGR* pathway amplification status cannot be excluded, because the population was smaller than expected.

**Trial registration:**

ClinicalTrials.gov identifier: NCT01528345. Registered 31 January 2012.

**Electronic supplementary material:**

The online version of this article (doi:10.1186/s13058-017-0807-8) contains supplementary material, which is available to authorized users.

## Background

Breast cancer is the most common type of cancer and the leading cause of cancer deaths in women worldwide [1]. In most countries, 3% to 12% of breast cancers are advanced or metastatic at diagnosis [2]. Most breast cancers are hormone receptor–positive (HR^+^), with 75% to 83% of breast cancers expressing estrogen receptor (ER)-α and/or progesterone receptor [3–5]. Likewise, approximately 86% to 87% of breast cancers are negative for overexpression of human epidermal growth factor receptor 2 (HER2^−^) [6, 7].

Currently, endocrine therapy is recommended as initial therapy for patients with HR^+^, HER2^−^ advanced breast cancer [8, 9]. Aromatase inhibitors are the standard of care for postmenopausal patients [9]. However, only 20% to 40% of patients respond to first-line therapy, and approximately one-half of responders relapse within 8–14 months [10]. Most patients eventually relapse because currently available treatments are not curative [11]. Second-line endocrine therapy (e.g., fulvestrant, aromatase inhibitors, tamoxifen) is recommended following relapse, but the response is generally short-lived. For example, the duration of response (DOR) to second-line fulvestrant or exemestane is approximately 3–5 months [12].

Several mechanisms of endocrine therapy resistance have been described, including activation of receptor tyrosine kinases (e.g., fibroblast growth factor receptor [FGFR]) and their downstream signaling pathways (e.g., phosphoinositide 3-kinase [PI3K]/Akt/mechanistic target of rapamycin [mTOR]), as well as activation of the cyclin-dependent kinases 4 and 6 that regulate cell cycle progression [13]. Efforts to improve outcomes and reduce endocrine therapy resistance have led to the development of combination therapies that included targeted agents against these resistance pathways. Positive results from the phase III Breast Cancer Trials of Oral Everolimus 2 (BOLERO-2) trial led to the approval of everolimus, an mTOR inhibitor, in combination with second-line exemestane in postmenopausal women with HR^+^, HER2^−^ advanced breast cancer that progressed during prior nonsteroidal aromatase inhibitor therapy [14, 15]. Later, positive results from the Palbociclib: Ongoing Trials in the Management of Breast Cancer (PALOMA) studies led to the approval of palbociclib (a cyclin-dependent kinase 4 and 6 inhibitor) as first-line therapy in combination with letrozole and as second-line therapy in combination with fulvestrant [16, 17].

Aberrant regulation of fibroblast growth factor (FGF) and FGFR signaling is associated with tumorigenic activity [18], an increased risk of developing breast cancer [19–21], and resistance to endocrine therapy [13]. Amplifications in *FGFR1* and *FGFR4* are found in 9% to 10% and 10% of primary breast cancers overall, respectively [22–25]. *FGFR1* amplification is more frequently associated with luminal B cancer, whereas *FGFR4* amplification is more common in HR^+^ tumors and a subset of HER2^+^ tumors [22, 26, 27]. *FGFR2* amplification is present in 4% of triple-negative breast cancers [28]. Overexpression of *FGFR* family members is associated with poor prognosis, including reduced overall survival (OS), disease-free survival, and relapse-free survival [24, 29–31]. FGFR overexpression is also associated with resistance to hormone therapy [26, 32] and chemotherapy [33, 34]. Importantly, FGFR1-induced tamoxifen resistance can be reversed by inhibiting *FGFR1* expression [32]. Aberrant PI3K/Akt/mTOR signaling is also seen in cells with *FGFR1* overexpression and amplification [26], and response to the PI3K inhibitor alpelisib is reduced in ER^+^/*PIK3CA*-mutant breast cancer cells that overexpress *FGFR1* [35]. Taken together, these results provide rationale for the investigation of FGFR inhibitors in breast cancer therapy.

Dovitinib (TKI258), a small-molecule inhibitor of FGFR1, FGFR2, and FGFR3 and other receptor tyrosine kinases [36], has shown preclinical activity in *FGFR*-expressing breast cancer models in vivo and in vitro [37]. Dovitinib inhibited cell proliferation in *FGFR*-amplified cell lines and showed antitumor activity in *FGFR*-amplified xenograft models [38]. In a phase II trial of single-agent dovitinib, encouraging clinical activity was observed in patients with HR^+^, HER2^−^ *FGF* pathway–amplified breast cancer [38]. *FGF* pathway amplification status was determined using in situ hybridization as part of the eligibility criteria (*FGFR1* only) and using quantitative polymerase chain reaction (qPCR) as an exploratory analysis (*FGFR1*, *FGFR2*, and *FGF3*). Correlative studies between *FGF* pathway amplification markers and antitumor activity indicated that dovitinib activity was higher in patients who had *FGF* pathway amplification measured by qPCR, particularly in those who had higher levels of *FGFR1* amplification (i.e., at least six copies of *FGFR1*) [38]. The combination of fulvestrant and dovitinib could potentially overcome resistance to endocrine therapy, thereby reducing the need for cytotoxic chemotherapy in relapsed patients. Together, these data and hypotheses prompted the initiation of this phase II, placebo-controlled trial of dovitinib plus fulvestrant in postmenopausal patients with HR^+^, HER2^−^ locally advanced or metastatic breast cancer.

The primary objective of this study was to determine the effect of treatment with dovitinib in combination with fulvestrant vs placebo plus fulvestrant on progression-free survival (PFS) in postmenopausal patients with HR^+^, HER2^−^ breast cancer that had progressed during or after prior endocrine therapy in all evaluable patients, regardless of *FGF* pathway amplification status, and in patients with *FGF* pathway amplification (as measured by qPCR using a cutoff of at least six copies of *FGFR1*, *FGFR2*, or *FGF3*). The key secondary objective was overall response rate (ORR). Additional secondary objectives included DOR, OS, safety, and pharmacokinetics of dovitinib.

## Methods

### Study design

We conducted a phase II, multicenter, international, randomized, double-blind, placebo-controlled trial (ClinicalTrials.gov identifier: NCT01528345) designed to evaluate the efficacy and safety of dovitinib in combination with fulvestrant in postmenopausal women with HR^+^, HER2^−^ locally advanced or metastatic breast cancer who had evidence of disease progression. Enrolled patients were randomized in a 1:1 ratio to receive dovitinib plus fulvestrant or placebo plus fulvestrant, stratified by *FGF* pathway amplification status (amplified vs nonamplified) and presence of visceral disease (yes vs no). All patients received fulvestrant 500 mg (intramuscular injection once every 4 weeks, with an additional dose 2 weeks after the initial dose) and dovitinib (500 mg) or placebo orally following a weekly 5 days on and 2 days off schedule until death, loss to follow-up, disease progression, or consent withdrawal.

Patients and medications were randomized using automated systems. At the time of initial screening, enrolled patients received a patient number, which was used as the primary identifier for the patient throughout the study. The interactive response technology provider generated a randomized patient list, using a validated automated system, by randomly assigning patient numbers to randomization numbers. Each randomization number was linked to a treatment arm and a medication number. Patients were randomized 1:1 to each of the study arms, with 45 *FGF* pathway–amplified and 30 *FGF* pathway–nonamplified patients planned in each arm. Medications were separately randomized by the study sponsor using a validated automated system that randomly assigned medication numbers to medication packs containing each of the study treatments. In this double-blind study, patients, investigators, study team members, and anyone involved in the conduct of the study remained blinded to the identity of the treatment from the time of randomization until database lock. The study medication and placebo had identical packaging, labeling, appearance, and administration schedules to conceal the identity of the treatments.

### Patients

Eligible patients were postmenopausal women with HR^+^, HER2^−^ locally advanced or metastatic breast cancer who had evidence of disease progression. Progression was defined as at least one measurable lesion per Response Evaluation Criteria In Solid Tumors (RECIST) version 1.1 or at least one nonmeasurable lytic or mixed bone lesion in the absence of measurable disease. Progression could have occurred  during or after prior endocrine therapy, within 12 months of the end of adjuvant endocrine therapy, or within 1 month of the end of any endocrine therapy for localized advanced or metastatic breast cancer. Eligible patients had confirmed postmenopausal status (i.e., aged ≥55 years with ≥1 year of amenorrhea, aged <55 years with ≥1 year of amenorrhea in the absence of ovarian suppression with an estradiol assay result of <20 pg/ml, or surgical menopause with bilateral oophorectomy), Eastern Cooperative Oncology Group (ECOG) performance status ≤2, and available archival or fresh tumor tissue for *FGF* pathway status determination in the primary tumor by the central laboratory. *FGF* pathway amplification was determined by a designated Clinical Laboratory Improvement Amendments–certified laboratory using a TaqMan PCR assay (Life Technologies, Carlsbad, CA, USA), as previously described [38]. Positive amplification for each *FGF* pathway marker tested (i.e., *FGFR1*, *FGFR2*, or *FGF3*) was defined as a copy number ≥6; copy number was quantified by comparison with a reference gene (human ribonuclease P RNA component H1) and calculated using CopyCaller software (version 1.1; Applied Biosystems, Foster City, CA, USA). Samples were considered to be *FGF* pathway–amplified if they had positive amplification of *FGFR1*, *FGFR2*, and/or *FGF3*. Given that the previously defined amplification cutoff of at least six copies for *FGFR1* was associated with higher sensitivity to dovitinib monotherapy [38], the same cutoff was used in this combination therapy study. Exclusion criteria included HER2 overexpression (assessed by immunohistochemistry), prior therapy with fulvestrant (as a single agent or in combination with other therapies) or FGFR inhibitors, or chemotherapy or more than one line of any prior hormone therapy for locally advanced or metastatic breast cancer. An estimated 1000 patients were expected to be screened for *FGF* pathway amplification in order to identify and randomize a total of 150 patients stratified by *FGF* pathway amplification and presence of visceral disease.

### Assessments

Radiographic assessments (computed tomography, magnetic resonance imaging, or radiography) were performed at screening, on day 5 of weeks 8 and 16, before fulvestrant administration every 8 weeks for the remainder of study treatment, and at the end of treatment (if not assessed within 8 weeks before visit). Safety assessments were performed continually until 30 days after the last study treatment. No additional tumor assessments were required to confirm response (complete response [CR] or partial response [PR]) outside the protocol-specified 8-week tumor assessment.

Patients who did not discontinue study treatment owing to disease progression or death, or who were not lost to follow-up or did not withdraw consent, were assessed every 8 weeks for disease status, ECOG performance status, and patient-reported outcomes until the start of new anticancer therapy, disease progression, death, loss to follow-up, or consent withdrawal. Survival follow-up was performed every 3 months until death, loss to follow-up, or consent withdrawal for patients who discontinued the treatment.

### Study endpoints

The coprimary endpoints were PFS in the overall patient population regardless of *FGF* pathway amplification status and PFS in the subgroup of patients with *FGF* pathway amplification. PFS was defined as the time from date of randomization to the date of first radiologically documented, investigator-assessed disease progression per RECIST v1.1 or death due to any cause. The key secondary endpoint was ORR, defined as the percentage of patients with best overall response of CR or PR. Additional secondary endpoints included DOR, OS, safety, and pharmacokinetics of dovitinib. Safety analysis, by treatment arm, was based on the frequency of adverse events (AEs), summarized by system organ class, severity (based on the Common Terminology Criteria for Adverse Events version 4.03), type, and relationship to study treatment.

### Analysis sets

The full analysis set, which consisted of all patients who were randomized and assigned study treatment, was the primary population for the efficacy endpoint analyses. The safety set consisted of all randomized patients who received at least one dose of any compound of the study treatment (dovitinib plus fulvestrant or placebo plus fulvestrant).

### Data analysis

The primary endpoint, PFS, was evaluated in each of the treatment arms using three sets of comparisons, following a Bayesian design: (1) all patients regardless of *FGF* pathway amplification status, (2) *FGF* pathway–amplified, and (3) *FGF* pathway–nonamplified (Additional file 1). Patients who did not have a PFS event at the time of analysis or who had received further antineoplastic therapy were censored at the time of the last tumor assessment. Kaplan-Meier plots were generated by treatment arm for the full population, *FGF* pathway–amplified subpopulation, and *FGF* pathway–nonamplified subpopulation. The HR of PFS in the full population was estimated using a Cox proportional hazards model stratified by *FGF* pathway amplification status and presence of visceral disease (yes vs no).

Efficacy of dovitinib plus fulvestrant over placebo plus fulvestrant was established if the estimated HR was <0.68 for the full population (i.e., improvement of approximately 3.0 months in median PFS) or <0.65 for the *FGF* pathway–amplified subpopulation (i.e., improvement of approximately 3.5 months in median PFS). Futility criteria in the *FGF* pathway–nonamplified subpopulation was determined if the posterior probability (HR >0.81) was >50% (i.e., improvement of <1.5 months in median PFS). The number of PFS events needed for the final analysis was calculated by assuming a 10% prevalence of *FGF* pathway amplification and a median PFS of 6.5 months with fulvestrant and placebo. To achieve the required number of PFS events for the final analysis (≥90 in the full population and ≥50 in the *FGF* pathway–amplified subgroup, whichever occurred later), a total of 150 patients had to be randomized as follows: 75 patients per treatment arm (45 *FGF* pathway–amplified and 30 *FGF* pathway–nonamplified).

Separate interim analyses were planned for patients with and without *FGF* pathway amplifications, owing to the faster enrollment expected for the *FGF* pathway–nonamplified subgroup. The first interim analyses occurred when 36 PFS events had been documented in the *FGF* pathway–nonamplified subgroup, and the second interim analysis occurred when ≥10 (20%) of 50 PFS events had been documented in the *FGF* pathway–amplified subgroup. The intent of these interim analyses was to assess the efficacy or futility of the study treatment. If the futility criteria were met (HR >0.81 in the first interim analysis; HR >0.7 in the second interim analysis), the study could be terminated early by the data monitoring committee.

The key secondary endpoint, ORR, was summarized as a percentage rate with 95% CI. OS was estimated using Kaplan-Meier analysis for each treatment arm; patients still alive at the time of analysis were censored at the last contact date.

## Results

### Patient demographics

**Fig. 1:**
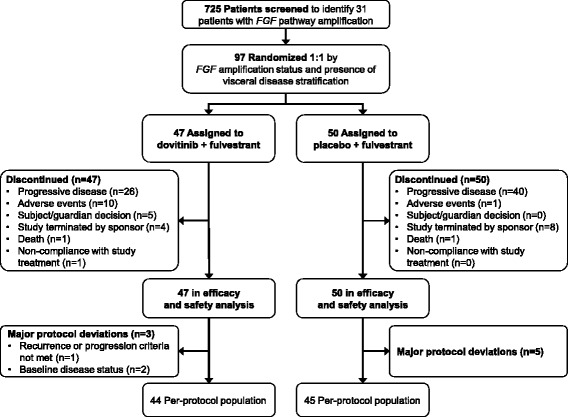
Consolidated Standards of Reporting Trials (CONSORT) diagram of patient disposition for patients randomized to receive dovitinib plus fulvestrant or placebo plus fulvestrant. *FGF* Fibroblast growth factor

From 15 May 2012 to 26 November 2014, a total of 97 postmenopausal patients with HR^+^, HER2^−^ locally advanced or metastatic breast cancer that had progressed during or after hormone therapy were enrolled in 36 centers. The last patient’s last visit was on 3 April 2015. All patients received fulvestrant; 47 were randomized to receive dovitinib and 50 were randomized to receive placebo (Fig. 1). Overall, 31 patients were classified as *FGF* pathway amplified (15 in the dovitinib arm vs 16 in the placebo arm); 725 patients were screened to enroll 31 *FGF* pathway–amplified patients. Although the data monitoring committee recommended continuing the study after 2 interim analyses, it subsequently recommended early termination of the study on 30 October 2014 due to lower than anticipated frequency of *FGF* pathway amplification and slow enrollment of patients with *FGF* pathway–amplified status. A total of 25 patients in each arm were classified as having visceral disease (53.2% vs 50.0% of patients in the dovitinib and placebo arms, respectively).

Baseline characteristics, including age, ECOG performance status, disease characteristics, and type and number of prior therapies were comparable between the study arms (Table 1). The median age for all patients was 63 (range 38–82) years; the median weight for all patients was 66.0 (range 38.0–135.5) kg; and the majority (57.7%) of patients had an ECOG performance status of 0. For patients with metastatic disease, the most common metastatic site was bone (77.3%), followed by lymph nodes (48.5%) and liver (39.2%). Most patients (76.3%) had been initially diagnosed ≥24 months before study start. Overall, 48.5% of patients had de novo stage IV disease, which was balanced between the dovitinib and placebo arms (48.9% vs 48.8% overall; 19.1% vs 16.0% *FGF* pathway–amplified; 29.8% vs 32.0% *FGF* pathway–nonamplified). The majority of patients relapsed at or within 12 months of the end of adjuvant treatment with any endocrine therapy (50 [51.5%] of 97 patients), and 48.5% (47 of 97) of patients progressed at or within 1 month of end of any endocrine therapy treatment for first-line treatment of metastatic disease. All patients had received prior antineoplastic therapy, including surgery (100%), hormone therapy (100%), radiotherapy (77.3%), and chemotherapy (66.0%). Approximately one-half (49.5%) of patients had received prior tamoxifen, and most patients had received prior aromatase inhibitors, including letrozole (42.3%), anastrozole (35.1%), and exemestane (17.5%). The majority (62.9%) of patients had received hormone therapy as their last prior treatment.

**Table 1:** Baseline patient demographics and disease characteristics

Baseline parameters	Fulvestrant + dovitinib (*n* = 47)	Fulvestrant + placebo (*n* = 50)	All patients (*n* = 97)
Patient demographics			
Median age (range), years	63 (44–82)	63 (38–82)	63 (38–82)
Median weight (range), kg	66.5 (38.0–95.0)	65.0 (41.0–135.5)	66.0 (38.0–135.5)
ECOG performance status, *n* (%)			
0	28 (59.6)	28 (56.0)	56 (57.7)
1	18 (38.8)	20 (40.0)	38 (39.2)
2	1 (2.1)	2 (4.0)	3 (3.1)
*FGF* pathway amplified, *n* (%)^a^			
No	32 (68.1)	33 (66.0)	65 (67.0)
Yes	15 (31.9)	17 (34.0)	32 (33.0)
Presence of visceral disease, *n* (%)^b^			
No	12 (25.5)	20 (40.0)	32 (33.0)
Yes	35 (74.5)	30 (60.0)	65 (67.0)
Disease characteristics, *n* (%)			
Primary site of cancer			
Breast	47 (100)	50 (100)	97 (100)
Metastatic site of cancer			
Bone	39 (83.0)	36 (72.0)	75 (77.3)
Lymph nodes	21 (44.7)	26 (52.0)	47 (48.5)
Liver	22 (46.8)	16 (32.0)	38 (39.2)
Other	19 (40.4)	8 (16.0)	27 (27.8)
Adrenal	3 (6.4)	3 (6.0)	6 (6.2)
Breast	0	1 (2.0)	1 (1.0)
Time from initial diagnosis of primary site to start of study drug			
<6 months	0	0	0
6 to <12 months	2 (4.3)	4 (8.0)	6 (6.2)
12 to <24 months	5 (10.6)	8 (16.0)	13 (13.4)
≥24 months	40 (85.1)	38 (76.0)	78 (80.4)
De novo stage IV	23 (48.9)	24 (48.0)	47 (48.5)
FGF pathway–amplified	9 (19.1)	6 (16.0)	
FGF pathway–nonamplified	14 (29.8)	16 (32.0)	
Prior therapies, *n* (%)			
Antineoplastic therapy^c^	47 (100)	50 (100)	97 (100)
Surgery	47 (100)	50 (100)	97 (100)
Hormone therapy	47 (100)	50 (100)	97 (100)
Radiotherapy	37 (78.7)	38 (76.0)	75 (77.3)
Chemotherapy	32 (68.1)	32 (64.0)	64 (66.0)
Therapy type at last treatment			
Hormone therapy	43 (91.5)	48 (96.0)	91 (93.8)
Chemotherapy	0	1 (2.0)	1 (1.0)
Other	4 (8.5)	1 (2.0)	5 (5.2)
Prior hormone therapies, *n* (%)			
Number of prior hormone regimens			
1	28 (59.6)	36 (72.0)	64 (66.0)
2	17 (36.2)	13 (26.0)	30 (30.9)
3	2 (4.3)	1 (2.0)	3 (3.1)
Setting^d^			
Adjuvant/neoadjuvant setting	38 (80.9)	37 (74.0)	75 (77.3)
Therapeutic setting	23 (48.9)	24 (48.0)	47 (48.5)
Prevention	4 (8.5)	3 (6.0)	7 (7.2)
Regimen type			
Tamoxifen	27 (57.4)	21 (42.0)	48 (49.5)
Letrozole	18 (38.3)	23 (46.0)	41 (42.3)
Anastrozole	16 (34.0)	18 (36.0)	34 (35.1)
Exemestane	8 (17.0)	9 (18.0)	17 (17.5)
Other^e^	1 (2.1)	4 (8.0)	5 (5.2)

*ECOG* European Cooperative Oncology Group, *FGF* Fibroblast growth factor

^a^Derived from biomarker data and determined by the central laboratory to be positive for gene amplification of fibroblast growth factor receptor 1 (*FGFR1*), *FGFR2*, or *FGF3*

^b^Based on electronic case report forms; *visceral* refers to lung, liver, pleural, or peritoneal involvement

^c^Includes patients who had medication, radiotherapy, or surgery

^d^A patient may have been treated in multiple settings

^e^Other prior hormone regimens included goserelin (*n* = 3), toremifene (*n* = 1), and triptorelin (*n* = 1)

### Efficacy

#### Progression-free survival

**Fig. 2:**
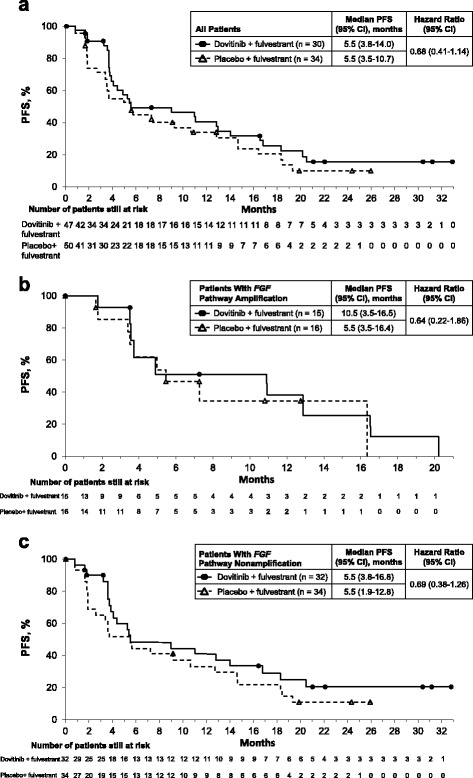
Investigator-assessed PFS by treatment for all patients (**a**), *FGF* pathway–amplified patients (**b**), and *FGF* pathway–nonamplified patients (**c**). *FGF* Fibroblast growth factor, *PFS* Progression-free survival

The median (95% CI) PFS for the full population were 5.5 (3.8–14.0) months and 5.5 (3.5–10.7) months in the dovitinib and placebo arms, respectively, with an estimated HR of 0.68 (95% CI 0.41–1.14) (Fig. 2a), with 30 events in the dovitinib arm and 34 events in the placebo arm. The estimated HR did not meet the criterion for superior efficacy of dovitinib vs placebo (i.e., HR <0.68). The median (95% CI) PFS values were 10.9 (3.5–16.5) months and 5.5 (3.5–16.4) months in the *FGF* pathway–amplified subgroup and 5.5 (3.8–16.8) months and 5.5 (1.9–12.8) months in the *FGF* pathway–nonamplified subgroup for the dovitinib and placebo arms, respectively (Fig. 2b and c). The HRs (95% CIs) were 0.64 (0.22–1.86) for the *FGF* pathway–amplified subgroup and 0.69 (0.38–1.26) for the *FGF* pathway–nonamplified subgroup, which was sufficient to meet the efficacy criteria for the *FGF* pathway–amplified subgroup (i.e., HR <0.65) and pass the futility criteria for the *FGF* pathway–nonamplified subgroup (i.e., HR >0.81). Fewer PFS events than expected occurred in *FGF* pathway–amplified patients (18 vs planned 50 events); thus, results in that subgroup should be interpreted with caution.

#### Best overall response

The ORRs (95% CIs) per local investigator assessment in all patients were 27.7% (15.6% to 42.6%) in the dovitinib arm and 10.0% (3.3% to 21.8%) in the placebo arm (Table 2). According to *FGF* pathway amplification status, the ORRs (95% CIs) for each treatment arm (dovitinib vs placebo) were 20.0% (4.3% to 48.1%) vs 12.5% (1.6% to 38.3%) in the *FGF* pathway–amplified subgroup and 31.3% (16.1% to 50.0%) vs 8.8% (1.9% to 23.7%) in the *FGF* pathway–nonamplified subgroup. In an exploratory analysis, we found no correlation between the *FGFR1* copy number and response to dovitinib in the *FGF* pathway–amplified subgroup, with the caveat that the analyzed patient population was small. When considering only those patients who had measurable disease at baseline (*n* = 41 [87.2%] in the dovitinib arm; *n* = 39 [78.0%] in the placebo arm), the ORRs (95% CIs) per local investigator assessment were 31.7% (18.1% to 48.1%) in the dovitinib arm and 12.8% (4.3% to 27.4%) in the placebo arm.

**Table 2:** Best overall response per local investigator review in the full analysis set

	Dovitinib + fulvestrant (*n* = 47)		Placebo + fulvestrant (*n* = 50)	
All patients				
Best overall response, *n* (%)				
CR	1 (2.1)		1 (2.0)	
PR	12 (25.5)		4 (8.0)	
SD	18 (38.3)		16 (32.0)	
PD	4 (8.5)		13 (26.0)	
Non-CR/non-PD	6 (12.8)		9 (18.0)	
Unknown	6 (12.8)		7 (14.0)	
Overall response rate (CR + PR) [95% CI], *n* (%)	13 (27.7) [15.6–42.6]		5 (10.0) [3.3–21.8]	
Median time to first response [95% CI], months	2.0 [1.5–18.8]		3.7 [1.6–9.1]	
Median duration of response [95% CI], months	13.5 [5.5–16.6]		14.7 [3.3–NE]	
Patients stratified by *FGF* pathway amplification	*FGF* pathway–amplified (*n* = 15)	*FGF* pathway–nonamplified (*n* = 32)	*FGF* pathway–amplified (*n* = 16)	*FGF* pathway–nonamplified (*n* = 34)
Best overall response, *n* (%)				
CR	0	1 (3.1)	1 (6.3)	0
PR	3 (20.0)	9 (28.1)	1 (6.3)	3 (8.8)
SD	7 (46.7)	11 (34.4)	4 (25.0)	12 (35.3)
PD	1 (6.7)	3 (9.4)	2 (12.5)	11 (32.4)
Non-CR/non-PD	2 (13.3)	4 (12.5)	6 (37.5)	3 (8.8)
Unknown	2 (13.3)	4 (12.5)	2 (12.5)	5 (14.7)
Overall response rate (CR + PR) [95% CI], *n* (%)	3 (20.0) [4.3–48.1]	10 (31.3) [16.1–50.0]	2 (12.5) [1.6–38.3]	3 (8.8) [1.9–23.7]
Median duration of response [95% CI], months	5.5 [3.2–16.3]	14.8 [5.5–NE]	14.7 [NE–NE]	10.9 [3.3–NE]

*Abbreviations: CR* Complete response, *FGF* Fibroblast growth factor, *NE* Not estimable, *PD* Progressive disease, *PR* Partial response, *SD* Stable disease

#### Time to response and duration of response

In patients who responded, the median (95% CI) time to first response in the dovitinib arm vs placebo arm was 2.0 (1.5–18.3) months vs 3.7 (1.6–9.1) months in the full population. Of the 13 patients who responded in the dovitinib arm, 11 patients (84.6%) responded within the first 4 months, 1 patient (7.7%) responded between 4 and <6 months, and 1 patient (7.7%) responded after 18 months of receiving the first dose of study treatment. Of the five patients who responded in the placebo arm, three patients (60.0%) responded within the first 4 months, and two patients (40.0%) responded between 6 and <12 months of initiating study treatment. The median (95% CI) values for DOR in the dovitinib arm vs placebo arm were 13.5 (5.5–16.6) months vs 14.7 (3.3–not estimable [NE]) months in the full population, 5.5 (3.2–16.3) months vs 14.7 (NE–NE) months in the *FGF* pathway–amplified subgroup, and 14.8 (5.5–NE) months vs 10.9 (3.3–NE) months in the *FGF* pathway–nonamplified subgroup. These data should be interpreted with caution owing to the small sample size.

#### Overall survival

**Fig. 3:**
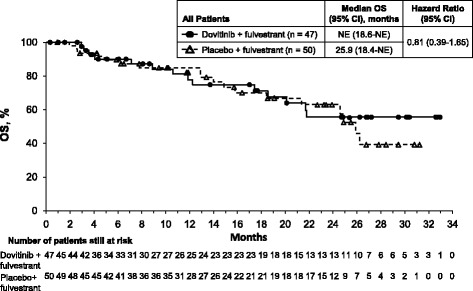
OS for all patients who received dovitinib plus fulvestrant or placebo plus fulvestrant. *NE* Not estimable, *OS* Overall survival

The median (95% CI) OS was not reached (18.6 months–NE) in the dovitinib arm and was 25.9 (18.4–NE) months in the placebo arm (Fig. 3).

### Safety

All patients received at least one dose of dovitinib or placebo and have discontinued study treatment (see Fig. 1). Of note, on the basis of the intention-to-treat principle, one patient with *FGF* pathway amplification who was misclassified as nonamplified at randomization remained in the nonamplified group in all analyses. The reasons for discontinuation in the dovitinib vs placebo arms were progressive disease (55.3% vs 80.0%), AEs (21.3% vs 2.0%), patient or guardian decision (10.6% vs 0%), termination by sponsor (8.5% vs 16.0%), death (2.1% vs 2.0%), and nonadherence to study treatment (2.1% vs 0%). The majority of patients who received dovitinib required at least one dose reduction or interruption (74.5% vs 26.0% for those who received placebo) and/or a dose change (57.4% vs 8.0% for those who received placebo); most patients required a dose interruption or delay or a dose change owing to experiencing an AE (70.2% and 53.2%, respectively) (Additional file 2: Table S1).

The most common any-grade AEs in the dovitinib arm, regardless of cause, were diarrhea (78.7%), nausea (72.3%), vomiting (57.4%), asthenia (38.8%), and headache (36.2%) (Table 3). In the placebo arm, diarrhea (32.0%), fatigue (26.0%), nausea and asthenia (22.0% each), and decreased appetite (16.0%) were the most common any-grade AEs. The most common grade 3 AEs (occurring in ≥10% of patients) in the dovitinib vs placebo arms were hypertension (21.3% vs 6.0%), diarrhea (14.9% vs 4.0%), alanine aminotransferase increase (14.9% vs 2.0%), fatigue (12.8% vs 2.0%), blood alkaline phosphatase increase (12.8% vs 0%), and γ-glutamyltransferase increase (10.6% vs 6.0%). The median (range) time to the onset of hypertension or blood pressure increase was 2.9 (0.1–28.1) weeks in the dovitinib arm and 2.7 (0.1–20.1) weeks in the placebo arm. In general, grade 4 AEs were infrequent and were comparable in the two arms, occurring in eight patients (17.0%) in the dovitinib arm and six patients (12.0%) in the placebo arm. Serious AEs suspected to be related to the study drug were reported in six patients (12.8%) in the dovitinib plus fulvestrant arm and included grade 3 pulmonary embolism, deep vein thrombosis, dehydration, esophageal varices hemorrhage, pneumonia, and varices esophageal (2.1% each), and grade 4 pulmonary embolism, ischemic cerebral infarction, and thrombocytopenia (2.1% each). In the placebo plus fulvestrant arm, two patients (4.0%) reported serious AEs suspected to be related to study drug (grade 4 hypotension [2.0%] and pancreatitis [2.0%]). The overall incidence of proteinuria and thyroid function abnormality was low in the dovitinib arm (two patients [4.3%] and one patient [2.1%], respectively; all grade 1/2 AEs) and was not reported in the placebo arm.

**Table 3:** Most common adverse events (occurring in ≥15% of patients), regardless of study drug relationship

Adverse events occurring in ≥ 15% of patients, by preferred term, *n* (%)	Dovitinib + fulvestrant (*n* = 47)		Placebo + fulvestrant (*n* = 50)	
	Any grade	Grade 3^a^	Any grade	Grade 3^a^
Any adverse event	47 (100)	32 (68.1)	47 (94.0)	19 (38.0)
Diarrhea	37 (78.7)	7 (14.9)	16 (32.0)	2 (4.0)
Nausea	34 (72.3)	4 (8.5)	11 (22.0)	1 (2.0)
Vomiting	27 (57.4)	3 (6.4)	4 (8.0)	0
Asthenia	18 (38.8)	4 (8.5)	11 (22.0)	1 (2.0)
Headache	17 (36.2)	2 (4.3)	3 (6.0)	0
Fatigue	16 (34.0)	6 (12.8)	13 (26.0)	1 (2.0)
Rash	16 (34.0)	1 (2.1)	3 (6.0)	0
Alanine aminotransferase increase	15 (31.9)	7 (14.9)	5 (10.0)	1 (2.0)
Dysgeusia	15 (31.9)	0	1 (2.0)	0
Decreased appetite	13 (27.7)	2 (4.3)	8 (16.0)	0
Hypertension	13 (27.7)	10 (21.3)	4 (8.0)	3 (6.0)
Dyspepsia	12 (25.5)	0	0	0
Blood alkaline phosphatase increase	11 (23.4)	6 (12.8)	1 (2.0)	0
Aspartate aminotransferase increase	10 (21.3)	3 (6.4)	4 (8.0)	1 (2.0)
Abdominal pain upper	10 (21.3)	0	3 (6.0)	0
Stomatitis	10 (21.3)	0	2 (4.0)	0
Anemia	9 (19.1)	2 (4.3)	4 (8.0)	1 (2.0)
γ-Glutamyltransferase increase	9 (19.1)	5 (10.6)^b^	4 (8.0)	3 (6.0)^b^
Pain in extremity	9 (19.1)	0	3 (6.0)	0
Dry skin	9 (19.1)	0	2 (4.0)	0
Dyspnea	8 (17.0)	1 (2.1)	6 (12.0)	0
Abdominal pain	8 (17.0)	0	5 (10.0)	0
Constipation	8 (17.0)	0	5 (10.0)	0
Hypertriglyceridemia	8 (17.0)	4 (8.5)	1 (2.0)	0

^a^Grade 4 adverse events occurred in eight patients (17.0%) in the dovitinib + fulvestrant arm and six patients (12.0%) in the placebo + fulvestrant arm, but no grade 4 adverse events were reported for any of the most common adverse events (occurring in ≥15% of patients), except where noted

^b^Grade 4 γ-glutamyltransferase increase was reported in three patients (6.4%) in the dovitinib + fulvestrant arm and one patient (2.0%) in the placebo + fulvestrant arm

More patients discontinued study treatment owing to AEs in the dovitinib arm than in the placebo arm (38.3% vs 8.0%). The most frequently reported AEs (occurring in ≥3% of patients) leading to discontinuation were diarrhea (6.4% vs 0%), alanine aminotransferase increase (4.3% vs 2.0%), aspartate aminotransferase increase (4.3% vs 2.0%), and rash (4.3% vs 0%) (Additional file 2: Table S2). A total of four on-treatment deaths were reported (two deaths in each treatment arm); of the two on-treatment deaths that occurred in the dovitinib arm, one patient died as a result of breast cancer progression and one patient died because of a pulmonary embolism suspected to be related to study treatment. Overall, 14 patients (29.8%) in the dovitinib arm and 18 patients (36.0%) in the placebo arm died during the entire study period (i.e., including the time beyond the 30-day end of treatment follow-up period).

## Discussion

In this randomized, double-blind trial, we evaluated the safety and efficacy of dovitinib plus fulvestrant compared with placebo plus fulvestrant in postmenopausal patients with HR^+^, HER2^−^ advanced breast cancer that progressed during or after prior endocrine therapy. The final analysis was initially planned to occur when 90 PFS events were recorded in the full population, including ≥50 PFS events in the *FGF*-amplified subgroup. However, the study was terminated early because of slow enrollment in the *FGF*-amplified subgroup.

In this study, patients in the *FGF* pathway–amplified subgroup who received dovitinib plus fulvestrant had prolonged median PFS (10.9 vs 5.5 months), with an estimated 36% risk reduction compared with patients who received placebo plus fulvestrant. However, a similar trend in risk reduction with dovitinib plus fulvestrant treatment (vs placebo plus fulvestrant) was seen in all patients (32%) and in patients without *FGF* pathway amplification (31%). This suggests that dovitinib plus fulvestrant may have antineoplastic activity regardless of *FGF* pathway amplification status in the evaluated patient population, although the estimated risk reduction reached statistical significance (as defined in the study protocol) only in the *FGF* pathway–amplified cohort. Furthermore, patients in the dovitinib plus fulvestrant arm had a higher ORR than patients in the placebo plus fulvestrant arm (27.7% vs 10.0%), regardless of *FGF* pathway amplification status (ORR 20.0% vs 12.5% in *FGF* pathway–amplified subgroup; ORR 31.3% vs 8.8% in *FGF* pathway–nonamplified subgroup). Nevertheless, these data should be interpreted cautiously. First, the small sample size in the *FGF* pathway–amplified subgroup contributed to a lower-than-expected number of PFS events and very large CIs. Second, we cannot exclude that dovitinib had an effect regardless of *FGF* pathway amplification status, given that the number of events for the full study population was 64 (30 in the dovitinib arm and 34 in the placebo arm), which was less than the 90 planned events. One potential explanation for the activity of dovitinib plus fulvestrant regardless of *FGF* pathway amplification status is that, as a multitargeted tyrosine kinase inhibitor, dovitinib targeted other pathways [36], such as signaling through vascular endothelial growth factor receptor or c-Kit, which are overexpressed in 10% to 11% and 11% to 17% of breast cancers, respectively [39, 40].

Safety data were consistent with the known safety profile of dovitinib [38, 41–44], with no new safety concerns identified with the use of dovitinib in combination with fulvestrant in patients with HR^+^, HER2^−^ advanced breast cancer. The use of FGFR inhibitors in breast cancer merits further investigation because other studies of single-agent FGFR inhibitors showed encouraging results in patients with breast cancer [38, 45–47]. Resistance to hormone therapy (i.e., tamoxifen) is potentially mediated by FGFR signaling through activation of the mitogen-activated protein kinase (MAPK) and PI3K pathways [26, 32]. For example, resistance to tamoxifen has been associated with constitutive activation of MAPK and the subsequent expression of cyclin D1 in *FGFR1*-amplified breast cancer cell lines [26]. Similarly, in ER^+^ cell lines, activation of *FGFR3* reduced sensitivity to tamoxifen and fulvestrant through activation of MAPK and PI3K signaling pathways [32]. Furthermore, the combination of dovitinib and the dual PI3K/mTOR inhibitor dactolisib (BEZ235) showed strong inhibition of PI3K pathway activation in vitro and in vivo, as well as antitumor activity in *FGFR*-expressing breast cancer models [48]. Currently, researchers in a phase Ib trial (ClinicalTrials.gov identifier: NCT01928459) are investigating the pan-FGFR inhibitor BGJ398 in combination with the selective PI3K inhibitor alpelisib (BYL719) in patients with solid tumors with *FGFR1*, *FGFR2*, and *FGFR3* alterations and phosphatidylinositol 4,5-bisphosphate 3-kinase catalytic subunit α mutations. Thus, further exploration of the use of FGFR inhibitors in combination with other agents is warranted.

The present study was terminated early because of slow accrual. The rates of *FGF* pathway amplification observed in this study were lower than previously reported. In the previous phase II monotherapy study, 10% of the patients screened who were enrolled in the study had *FGFR1* amplification [38], whereas approximately 5% of patients screened and randomized in this study had *FGF* pathway amplifications. In the phase II monotherapy study, many of the patients were prescreened by the French cooperative group, which reduced the overall number of patients needed to be screened. In addition, the eligibility criteria allowed more heavily pretreated patients to be enrolled, which expanded the pool of potential patients. Conducting clinical trials in molecularly selected patient populations is challenging, particularly because the screening failure rate is high with current trial designs [49], accrual can be slow when the molecular aberration is very rare, and patient dropout rates [50] and costs [51] can be high. In this study, accrual of *FGF*-amplified patients was very slow and resulted in the early termination of the trial, thereby confounding interpretation of the results. Several strategies have been proposed to overcome these challenges. A novel idea to increase rapid recruitment of patients with rare molecular markers is to develop molecular screening programs that screen several genes at a time in a large patient pool, using next-generation sequencing assays, and then to guide patients to specific clinical trials on the basis of their specific biomarkers [52, 53]. One example is the National Cancer Institute’s Molecular Analysis for Therapy Choice (NCI MATCH) trial (ClinicalTrials.gov identifier: NCT02465060); patients are screened for approximately 200 genes, assigned to a study arm on the basis of a molecular abnormality, and followed for response and PFS [52]. The NCI MATCH study currently includes 24 arms, in one of which investigators are evaluating the FGFR inhibitor AZD4547 in patients with FGFR pathway aberrations (*FGFR1–FGFR3* amplification, mutation, or translocation). Following progression, patients may be rescreened and enrolled in a second study arm; patients may also receive their screening results and decide, together with their doctor, to receive alternative therapy [52]. It remains to be seen whether new trial designs will have widespread support [50].

## Conclusions

In this placebo-controlled study of dovitinib in combination with fulvestrant in postmenopausal patients with HR^+^, HER2^−^ advanced or metastatic breast cancer that progressed during or after prior endocrine therapy did not identify any new safety findings. Dovitinib in combination with fulvestrant showed promising clinical activity in the *FGF* pathway*–*amplified subgroup. However, the reported data should be interpreted with caution, given that fewer PFS events than expected occurred in the *FGF* pathway–amplified patients and that we cannot exclude an effect of dovitinib regardless of *FGR* pathway amplification status, owing to the smaller-than-expected sample size.

## Additional files

Additional file 1: Figure S1. Primary endpoint data analysis. (PDF 163 kb)
Additional file 2: Table S1. Dose changes and dose delays by treatment arm. **Table S2.** Adverse events leading to study drug discontinuation regardless of study drug relationship. **Table S3.** Study center. (DOCX 17 kb)

## Abbreviations

AE: Adverse event
CONSORT: Consolidated Standards of Reporting Trials
CR: Complete response
DOR: Duration of response
ECOG: Eastern Cooperative Oncology Group
ER: Estrogen receptor
ET: Endocrine therapy
FGF: Fibroblast growth factor
FGFR: Fibroblast growth factor receptor
HER2: Human epidermal growth factor receptor 2
HR: Hormone receptor
MAPK: Mitogen-activated protein kinase
mTOR: Mechanistic target of rapamycin
NCI MATCH: National Cancer Institute’s Molecular Analysis for Therapy Choice trial
NE: Not estimable
ORR: Overall response rate
OS: Overall survival
PCR: Polymerase chain reaction
PD: Progressive disease
PFS: Progression-free survival
PI3K: Phosphoinositide 3-kinase
PR: Partial response
qPCR: Quantitative polymerase chain reaction
RECIST: Response Evaluation Criteria In Solid Tumors
SD: Stable disease
