# Supplementary material for: Phase II, randomized, placebo-controlled study of dovitinib in combination with fulvestrant in postmenopausal patients with HR+, HER2− breast cancer that had progressed during or after prior endocrine therapy
Source: Breast Cancer Res. 2017 Feb 10;19:18. doi: 10.1186/s13058-017-0807-8 (PMC5301372; doi:10.1186/s13058-017-0807-8)
Supplement: Additional file 1: Figure S1. — Primary endpoint data analysis. (PDF 163 kb) [file 13058_2017_807_MOESM1_ESM.pdf]

Figure S1. Data analysis

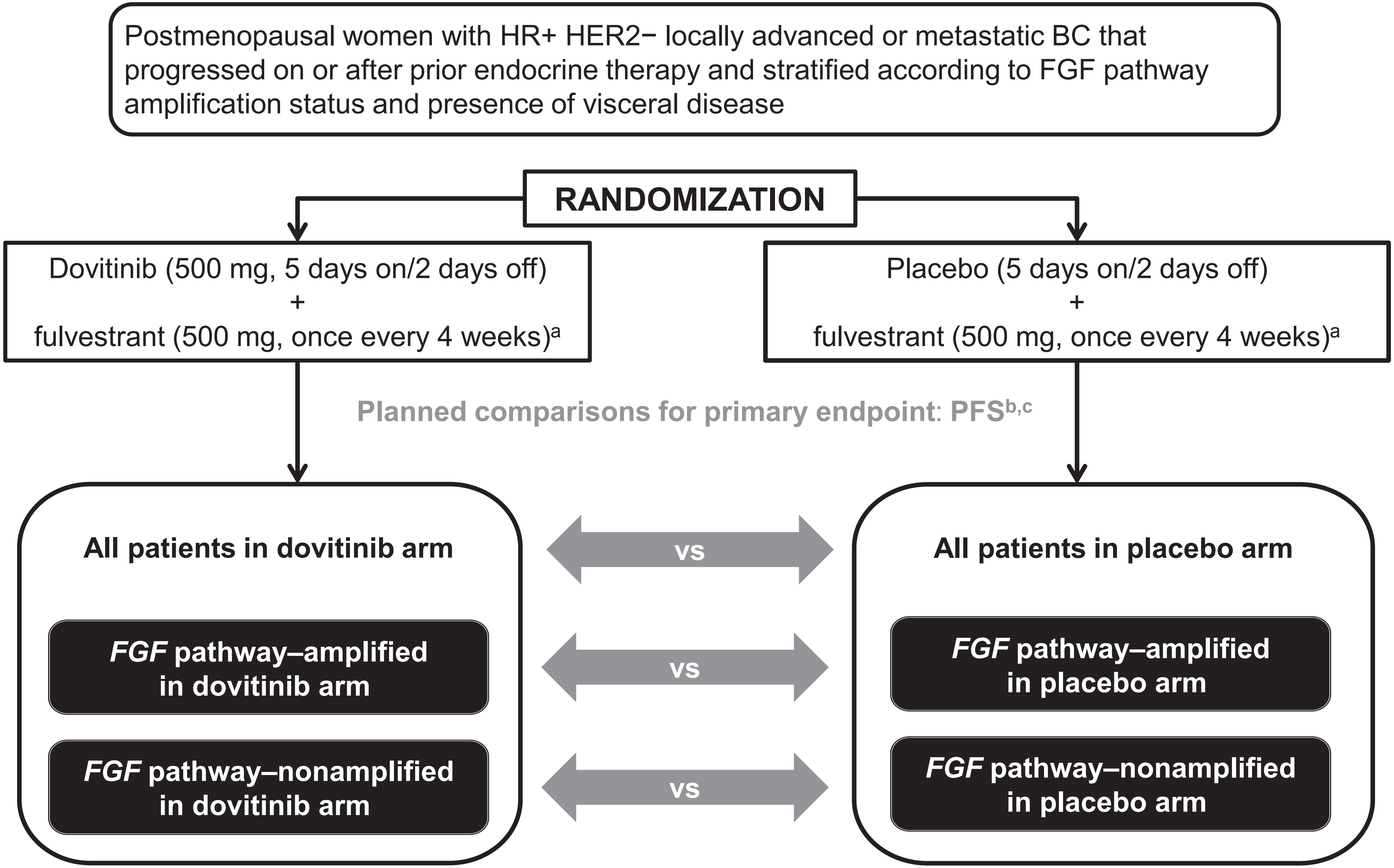

[a] Fulvestrant was given once every 4 weeks with one additional dose 2 weeks after the initial dose

[b] Survival follow-up data to be collected until 1 year after final data analysis data-cutoff data or until all patients discontinued treatment, whichever comes first.

[c] Final analysis planned after ≥90 PFS events, including ≥50 events from *FGF*-amplified patients.

FGF, fibroblast growth factor; HER2, human epidermal growth factor receptor 2; HR, hormone receptor; PFS, progression-free survival.
