# Supplementary material for: Phase II, randomized, placebo-controlled study of dovitinib in combination with fulvestrant in postmenopausal patients with HR+, HER2− breast cancer that had progressed during or after prior endocrine therapy
Source: Breast Cancer Res. 2017 Feb 10;19:18. doi: 10.1186/s13058-017-0807-8 (PMC5301372; doi:10.1186/s13058-017-0807-8)
Supplement: Additional file 2: Table S1. — Dose changes and dose delays by treatment arm. Table S2. Adverse events leading to study drug discontinuation regardless of study drug relationship. Table S3. Study center. (DOCX 17 kb) [file 13058_2017_807_MOESM2_ESM.docx]

**Table S1. Dose changes and dose delays by treatment arm**

|  | **Dovitinib + fulvestrant**  **(n = 47)** | | **Placebo + fulvestrant**  **(n = 50)** | |
| --- | --- | --- | --- | --- |
|  | **Dovitinib** | **Fulvestrant** | **Placebo** | **Fulvestrant** |
| Patients requiring dose interruption/delay, n (%) |  |  |  |  |
| Overall | 35 (74.5) | 1 (2.1) | 13 (26.0) | 3 (6.0) |
| 1 delay/interruption | 17 (36.2) | 1 (2.1) | 4 (8.0) | 3 (6.0) |
| ≥ 1 delay/interruption | 18 (38.3) | 0 | 9 (18.0) | 0 |
| Reasons for dose interruption, n (%) |  |  |  |  |
| Adverse event | 33 (70.2) | 1 (2.1) | 11 (22.0) | 2 (4.0) |
| Dosing error | 2 (4.3) | 0 | 4 (8.0) | 0 |
| Physician decision | 3 (6.4) | 0 | 2 (4.0) | 1 (2.0) |
| Patient/guardian decision | 5 (10.6) | 0 | 2 (4.0) | 0 |
| Technical problems | 2 (4.3) | 0 | 1 (2.0) | 0 |
| Patients requiring dose change, n (%) |  |  |  |  |
| Overall | 27 (57.4) | 0 | 4 (8.0) | 0 |
| 1 dose change | 10 (21.3) | 0 | 2 (4.0) | 0 |
| ≥ 1 dose change | 17 (36.2) | 0 | 2 (4.0) | 0 |
| Reasons for dose change, n (%) |  |  |  |  |
| Adverse event | 25 (53.2) | 0 | 2 (4.0) | 0 |
| Dosing error | 3 (6.4) | 0 | 2 (4.0) | 0 |
| Physician decision | 2 (4.3) | 0 | 0 | 0 |

Table S2. Adverse events leading to study drug discontinuation regardless of study drug relationship

|  | **Dovitinib + fulvestrant**  **(n = 47)** | **Placebo + fulvestrant**  **(n = 50)** |
| --- | --- | --- |
| Any adverse event leading to discontinuation | 18 (38.3) | 4 (8.0) |
| Diarrhea | 3 (6.4) | 0 |
| Alanine aminotransferase increase | 2 (4.3) | 1 (2.0) |
| Aspartate aminotransferase increase | 2 (4.3) | 1 (2.0) |
| Rash | 2 (4.3) | 0 |
| Pericardial effusion | 1 (2.1) | 0 |
| Eye pain | 1 (2.1) | 0 |
| Visual impairment | 1 (2.1) | 0 |
| Abdominal pain | 1 (2.1) | 0 |
| Gastritis | 1 (2.1) | 0 |
| Gastrointestinal motility disorder | 1 (2.1) | 0 |
| Nausea | 1 (2.1) | 0 |
| Vomiting | 1 (2.1) | 0 |
| γ-glutamyltransferase increase | 1 (2.1) | 0 |
| Arthritis | 1 (2.1) | 0 |
| Bone pain | 1 (2.1) | 0 |
| Pain in extremity | 1 (2.1) | 0 |
| Ischemic cerebral infarction | 1 (2.1) | 0 |
| Dyspnea | 1 (2.1) | 0 |
| Pulmonary embolism | 1 (2.1) | 0 |
| Dermatitis | 1 (2.1) | 0 |
| Dermatitis acneiform | 1 (2.1) | 0 |
| Milia | 1 (2.1) | 0 |
| Pigmentation disorder | 1 (2.1) | 0 |
| Skin toxicity | 1 (2.1) | 0 |
| Deep vein thrombosis | 1 (2.1) | 0 |
| Tooth abscess | 0 | 1 (2.0) |
| Blood bilirubin increase | 0 | 1 (2.0) |
| Bone lesion | 0 | 1 (2.0) |
| Osteonecrosis of jaw | 0 | 1 (2.0) |
| Laryngospasm | 0 | 1 (2.0) |

Table S3. Study center

| **Country** | **Study center ethics committee** |
| --- | --- |
| Argentina | Sanatorio de la Providencia, Buenos Aires |
|  | Céntro Médico San Roque, Tucurama |
| Austria | Allgemeines Krankenhaus der Stadt Wien—Universität Klinik für Innere Medizin III, Wien |
|  | Landeskrankenhaus Salzburg—Universität Klinikum der Paracelsus Medizinishe Privatuniversität, Salzburg |
| Belgium | Gasthuisberg University Hospital, Leuven |
|  | Sint-Augustinus Ziekernhuis, Wilrijk |
| Brazil | Hospital do Câncer de Londrina, Londrina |
| France | Gustave Roussy, Villejuif |
|  | Centre René Gauducheau, Saint-Herblain |
|  | Institut Bergoine, Bordeaux |
|  | CHU de Besançon, Besançon |
| Italy | Azienda Ospedaliero-Universitaria di Parma, Parma |
|  | Presidio Ospedaliero di Sondrio—Azienda Ospedaliera Della Valtellina e Valchiavenna, Sondrio |
|  | Presidio Ospedaliero di Macerata—Area Vasta Azienda Sanitaria Unica Regionale |
| Hungary | Magyar Hnvedseg Egeszsegugyi Kospont, Budapest |
|  | Petz Aladar Megyei Oktato Korhaz, Gyor |
|  | Szent-Györgyi Albert Klinikai Központ—Onkotérapiás Klinika, Szeged |
|  | Jász-Nagykun-Szolnok Megyei Hetenyi Geza Korhaz-Rendelointezet, Szolnok |
| The Netherlands | Universitair Medisch Centrum, Maastricht |
|  | Erasmus Medisch Centrum, Rotterdam |
| Russia | N. N. Petrov Research Institute of Oncology, St Petersburg |
|  | Ryazan Regional Clinical Oncological Dispensary, Ryazan |
| Spain | Hospital Vall d’Hebron, Barcelona |
| Taiwan | National Taiwan University Hospital, Taipei |
|  | Taichung Veterangs General Hospital, Taichung |
|  | Chang Gung Memorial Hospital, Niasong Township |
| United States | Highlands Oncology Group, Fayetteville, AR |
|  | University of California San Diego, La Jolla, CA |
|  | Oncology Specialists, SC, Niles, IL |
|  | Moffitt Cancer Center, Tampa, FL |
|  | Cedar Sinai Medical Center, Los Angeles, CA |
|  | City of Hope National Medical Center, Duarte, CA |
|  | Virginia Cancer Specialists, PC, Fairfax, VA |
|  | Cancer Centers of the Carolinas, Greenville, SC |
|  | Medical Oncology Associates, PS, Spokane, WA |
